# *Vital Signs:* Epidemiology and Recent Trends in Methicillin-Resistant and in Methicillin-Susceptible *Staphylococcus aureus* Bloodstream Infections — United States

**DOI:** 10.15585/mmwr.mm6809e1

**Published:** 2019-03-08

**Authors:** Athena P. Kourtis, Kelly Hatfield, James Baggs, Yi Mu, Isaac See, Erin Epson, Joelle Nadle, Marion A. Kainer, Ghinwa Dumyati, Susan Petit, Susan M. Ray, Emerging Infections Program MRSA author group, David Ham, Catherine Capers, Heather Ewing, Nicole Coffin, L. Clifford McDonald, John Jernigan, Denise Cardo

**Affiliations:** ^1^Division of Healthcare Quality Promotion, National Center for Emerging and Zoonotic Infectious Diseases, CDC; ^2^California Department of Health; ^3^California Emerging Infections Program, Oakland, California; ^4^Tennessee Emerging Infections Program, Nashville, Tennessee; ^5^New York–Rochester Emerging Infections Program, University of Rochester Medical Center, Rochester, New York; ^6^Connecticut Emerging Infections Program, New Haven, Connecticut; ^7^Georgia Emerging Infections Program, Atlanta Georgia.

## Abstract

**Introduction:**

*Staphylococcus aureus* is one of the most common pathogens in health care facilities and in the community, and can cause invasive infections, sepsis, and death. Despite progress in preventing methicillin-resistant *S. aureus* (MRSA) infections in health care settings, assessment of the problem in both health care and community settings is needed. Further, the epidemiology of methicillin-susceptible *S. aureus* (MSSA) infections is not well described at the national level.

**Methods:**

Data from the Emerging Infections Program (EIP) MRSA population surveillance (2005–2016) and from the Premier and Cerner Electronic Health Record databases (2012–2017) were analyzed to describe trends in incidence of hospital-onset and community-onset MRSA and MSSA bloodstream infections and to estimate the overall incidence of *S. aureus* bloodstream infections in the United States and associated in-hospital mortality.

**Results:**

In 2017, an estimated 119,247 *S. aureus* bloodstream infections with 19,832 associated deaths occurred. During 2005–2012 rates of hospital-onset MRSA bloodstream infection decreased by 17.1% annually, but the decline slowed during 2013–2016. Community-onset MRSA declined less markedly (6.9% annually during 2005–2016), mostly related to declines in health care–associated infections. Hospital-onset MSSA has not significantly changed (p = 0.11), and community-onset MSSA infections have slightly increased (3.9% per year, p<0.0001) from 2012 to 2017.

**Conclusions and Implications for Public Health Practice:**

Despite reductions in incidence of MRSA bloodstream infections since 2005, *S. aureus* infections account for significant morbidity and mortality in the United States. To reduce the incidence of these infections further, health care facilities should take steps to fully implement CDC recommendations for prevention of device- and procedure-associated infections and for interruption of transmission. New and novel prevention strategies are also needed.

*On March 5, 2019, this report was posted as an *MMWR* Early Release on the *MMWR* website (*https://www.cdc.gov/mmwr*).*

## Introduction

*Staphylococcus aureus* is a major cause of community- and health care–associated infections (*1*), ranging from superficial skin and soft tissue infections (SSTI) to invasive infections, sepsis, and death. Methicillin-resistant *S. aureus* (MRSA) has long been recognized as a pathogen associated with health care settings; however, in the 1990s, community-associated MRSA infections, causing mostly SSTI, emerged in the United States (*2*). Substantial progress has been achieved in preventing MRSA bloodstream infections in U.S. health care facilities (*3*–*5*) after widespread introduction of enhanced infection control efforts in acute-care hospitals.

Although the rates of hospital-onset MRSA bloodstream infections have substantially decreased, evidence from the National Healthcare Safety Network (NHSN) and from the Emerging Infections Program (EIP) surveillance system suggests that the decline might have slowed in more recent years (*4*,*6*); the United States is not on track to meet the 2020 goal of the Healthcare-Associated Infection National Action Plan of a 50% reduction in hospital-onset MRSA bloodstream infections from the 2015 baseline (*7*). Moreover, to protect patients, expanded efforts are needed to prevent methicillin-susceptible *S. aureus* (MSSA), which causes approximately half of all health care–associated *S. aureus* infections (*8*). There is little information on the current epidemiology of MSSA infections in the United States, and available data might not be nationally representative (*9*–*11*).

A critical assessment of recent trends and incidence of both MRSA and MSSA invasive disease in the United States is crucial to informing public health policy and formulating a framework of approaches to further prevent *S. aureus* infections. In this report, recent data from the EIP population surveillance and two large electronic health record (EHR) data sets from over 400 U.S. acute care hospitals were used to update estimates of MRSA and MSSA bloodstream infections, and to estimate associated in-hospital mortality.

## Methods

**EIP population MRSA surveillance.** MRSA bloodstream infection data were obtained from CDC’s EIP active laboratory- and population-based surveillance for invasive MRSA in selected counties from six sites* reporting data continually from 2005 to 2016 (population in 2016 = 13 million). A case of MRSA bloodstream infection was defined as isolation of MRSA from a blood culture in a resident of the catchment area, who had not had a positive invasive culture from a normally sterile site in the preceding 30 days. Annual incidence was calculated per 100,000 census population and stratified according to patient epidemiologic exposure, determined through medical record review as 1) hospital-onset if the culture was obtained on or after the fourth day of an inpatient hospitalization; 2) health care–associated community-onset, if the culture was obtained from an outpatient or during the first 3 days of hospitalization in a patient with one of several significant prior health care exposures; and 3) community-associated, otherwise. Community-onset infections comprise health care–associated community-onset and community-associated infections. Further details about the surveillance program can be found elsewhere (*3*). Adjusted annual decreases were modeled using Poisson regression and accounting for changes in the overall population and dialysis population demographics. Postcensus bridged-race census files were used for EIP analyses.

**EHR databases.** The Premier Healthcare Database (*12*) and Cerner Health Facts EMR (*13*) data were used to identify *S. aureus* bloodstream infections among patients discharged from participating acute care hospitals reporting results of microbiologic cultures with antimicrobial susceptibility testing during January 1, 2012–December 31, 2017. A case was defined as the identification of *S. aureus* in a blood culture with reported antimicrobial sensitivity, without a positive *S. aureus* blood culture in the preceding 14 days. Community-onset and hospital-onset cases were defined as for EIP surveillance. Incidences of MSSA and MRSA were calculated as the number of community-onset cases per 1,000 hospital discharges and the number of hospital-onset cases per 10,000 patient-days. Trends in monthly incidence during 2012–2017 were assessed using generalized estimating equations to fit negative binomial regression models adjusted for seasonality and certain hospital characteristics and accounting for clustering and repeated measures. The outcome variable was the number of *S. aureus* bloodstream infections, and the predictor variable was a continuous time covariate. Adjusted rates are presented as relative annual trends. Deaths associated with *S. aureus* bloodstream infections were defined as deaths or discharges to hospice for patient hospitalizations with documented *S. aureus* bloodstream infections. Differences in annual mortality were assessed using generalized estimating equations binomial models adjusting for all the same characteristics as for incidence. Hospital characteristics were used in a raking-procedure to determine weights to extrapolate the number of discharges included in the sample to match the distribution of discharges for all hospitals from the American Hospital Association survey (*14*). Using these weights, national estimates of cases of *S. aureus* bloodstream infections and deaths associated with *S. aureus* bloodstream infections were extrapolated. All statistical analyses were performed using SAS (version 9.4; SAS Institute).

## Results

**FIGURE 1:**
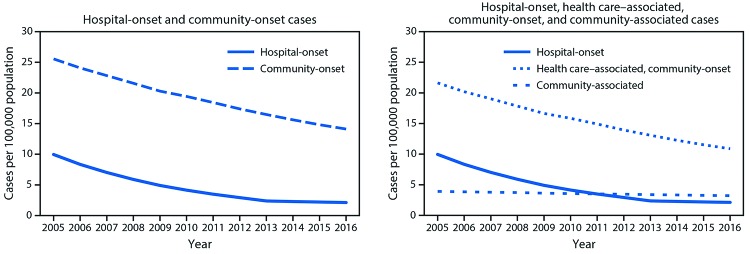
Adjusted* methicillin-resistant *Staphylococcus* *aureus* bloodstream infection rates from population based surveillance — six U.S. Emerging Infections Program sites,^†^ 2005–2016 * Adjusted for year and distribution of age, sex, and race among overall and dialysis population. Community-onset infections comprise health care–associated community-onset and community-associated infections ^†^ California (three counties), Connecticut (statewide), Georgia (eight counties), Minnesota (one county), New York (one county), and Tennessee (one county).

**Rates of hospital-onset and community-onset MRSA, EIP surveillance, 2005–2016.** From 2005 to 2016, the incidence of hospital-onset and community-onset MRSA bloodstream infection declined 74% and 40%, respectively (Figure 1). The decline in hospital-onset MRSA bloodstream infection rates has slowed in more recent years: adjusted rates decreased by 17.1% per year (p<0.001) during 2005–2012 but did not significantly change during 2013–2016 (p = 0.25). Adjusted community-onset MRSA bloodstream infection rates declined by 6.9% per year during 2005–2016 (p<0.001). Declines in rates of health care–associated community-onset infections accounted for most of the decline in community-onset MRSA bloodstream infections during 2005–2016 (Figure 1). Adjusted health care–associated community-onset bloodstream infection rates declined by 7.8% per year (p = 0.001), but community-associated bloodstream infections declined by only 2.5% per year (p = 0.001).

**FIGURE 2:**
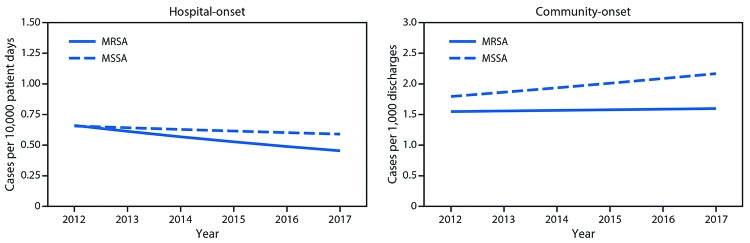
Adjusted* hospital-onset and community-onset rates of *Staphylococcus aureus* bloodstream infections — Premier and Cerner Hospitals, United States, 2012–2017 **Abbreviations:** MRSA = methicillin-resistant *Staphylococcus aureus*; MSSA = methicillin-susceptible *Staphylococcus aureus*. * Modeled relative to observed rates in 2012. Model adjusts for discharge month and year and hospital region, teaching status, bed size, and distributions of patient age, sex and race, in addition to accounting for repeated measures and clustering by facility.

**Rates of hospital-onset and community-onset MRSA and MSSA and associated mortality, EHR data, 2012–2017.** From 2012 to 2017, 447 hospitals contributed data (average per year = 325). During this time, adjusted hospital-onset MRSA bloodstream infection rates declined 7.3% per year (p<0.0001) (Figure 2), with no significant change in community-onset MRSA rates (p = 0.35). Hospital-onset MSSA rates did not change (p = 0.11), and community-onset MSSA rates significantly increased (3.9% per year, p<0.001) (Figure 2).

The overall unadjusted in-hospital mortality among patients with *S. aureus* bloodstream infections over the study period was 18%. No significant change was observed from 2012 to 2017, although significant differences were observed by epidemiologic classification: unadjusted MRSA and MSSA mortality rates were higher for hospital-onset cases (29% and 24%, respectively) than for community-onset cases (18% and 14%, respectively) (p<0.001).

**Estimated morbidity of *S. aureus* bloodstream infections and in-hospital mortality, EHR data, United States, 2017.** Overall, an estimated 119,247 cases of *S. aureus* bloodstream infections and 19,832 associated deaths occurred nationwide in 2017.

## Conclusions and Comments

This study identified substantial reductions in hospital-onset MRSA bloodstream infection rates between 2005 and 2012; however, since 2012, the rate of decline has slowed. These trends are consistent with recent data from NHSN (*4*). Less marked declines were noted in rates of community-onset MRSA bloodstream infections compared with those of hospital-onset infections. The detailed epidemiologic information that EIP collects allowed subclassification of community-onset MRSA infections into those with prior health care exposure (health care–associated community-onset), which account for the majority of cases, and those without health care exposure (community-associated). Most of the reduction in MRSA bloodstream infection is attributable to reductions in health care–associated MRSA. Community-associated MRSA infection rates have changed little overall. Hospital-onset MSSA infection rates have not changed since 2012, whereas community-onset-MSSA infection rates might be increasing slightly.

The reasons for the declines in hospital-onset MRSA bloodstream infections might be attributable to a variety of infection control efforts, including improvements in preventing device- and procedure-associated infections (*15*–*17*), as well as efforts to interrupt MRSA transmission in the hospital setting (*5*,*18*). As has been reported previously (*17*), significant national reductions in central-line–associated bloodstream infections occurred during 2001–2009, particularly in those caused by *S. aureus*; these reductions have continued through more recent years (*4*). Meanwhile, evidence from the National Veterans Affairs system suggests that decreasing hospital transmission of MRSA likely also contributed to the observed reductions (*19*,*20*).

National MRSA reductions primarily reflect declines in the incidence of infections caused by USA100 strains, which are predominantly transmitted in health care settings, and, to a lesser extent, USA300 strains, which are predominantly transmitted in the community (*21*). Historically, large shifts in *S. aureus* strain epidemiology have occurred (*22*). Whereas the reasons for some of these shifts might be related to strain virulence and fitness, health care–related interventions are likely to have played a role in the decrease in USA100.

The recent slowing in the reduction in hospital-onset MRSA bloodstream infections and the limited decline in community-associated MRSA and in MSSA infections point to the need for an updated *S. aureus* prevention framework, including greater use of evidence-based practices that can reduce transmission and prevent device- and procedure-associated infections, as well as new and novel approaches. These include strategies to suppress *S. aureus* colonization in patients during periods of high risk for invasive *S. aureus* infection, such as when invasive devices are in place, during admission to high-risk hospital units, or perioperatively for certain high-risk surgical procedures. The experience with MRSA suggests that the postdischarge period might also be important for targeting innovative prevention efforts: EIP data suggest that the majority of all MRSA bloodstream infections are health care–associated community-onset, and most occur in the 3 months after hospital discharge (*3*). A recent study suggests that prescribing serial decolonization protocols at the time of hospital discharge could significantly reduce postdischarge *S. aureus* infections (*23*). Suppression of *S. aureus* colonization might play an important role in decreasing transmission, but data to recommend this approach as a replacement for currently recommended strategies to prevent transmission, such as contact precautions, are insufficient (*24*). All hospitals should have strategies in place for preventing *S. aureus* infections; however, the prevention impact might be greatest in those with a particularly high *S. aureus* incidence. A recent review of NHSN data indicated that a relatively small number of hospitals (approximately 200) account for slightly over half of the hospital-onset MRSA incidence in excess of the 2020 goals and could be prioritized for prevention to reduce MRSA bloodstream infections nationally (NHSN, unpublished data). 

 Community-associated MRSA infections provide a reservoir that contributes to health care–associated disease incidence and fuels transmission both outside and within health care settings. USA300 strains, for example, emerged in the community and spread to health care settings (*25*). Community-associated *S. aureus* infections are not declining, and the ongoing opioid epidemic might be contributing to this trend. Emerging evidence suggests a 16-fold risk for invasive MRSA infection among persons who inject drugs; 9.2% of invasive MRSA cases in 2016 occurred in persons who inject drugs (*26*). Prevention of opioid misuse, increasing access and linkage to medication-assisted treatment for persons with opioid use disorder (*27*), ensuring access to sterile injecting equipment, improving education about safer injection practices and how to recognize early signs of infection, and linking those with an infection to care are needed. Additionally, community-associated *S. aureus* infections are known to disproportionately affect persons in lower socioeconomic strata (*28*); this has implications for the formulation of approaches to enhance prevention. The observed increases in rates of community-onset MSSA infections highlight the need to systematically study the epidemiology of MSSA and develop innovative, evidence-based prevention strategies for this setting. Research for a vaccine or for novel ways to decrease *S. aureus* bioburden should continue.

The incidence of *S. aureus* bloodstream infections and associated deaths is substantial and consistent with estimates using Nationwide Inpatient Sample data (*29*). Mortality was unchanged over the time studied and comparable to what was achieved in the VA hospital system through implementation of improved clinical management of infection (*28*). Appropriate and timely diagnosis and antimicrobial susceptibility-guided treatment of *S. aureus* infections remain key to reducing poor outcomes and preventing sepsis and death (*29*).

The findings in this report are subject to at least two limitations. First, the lack of detailed epidemiologic information on previous health care exposures captured in EHR precluded subclassification of community-onset infections into those with and without previous health care exposures. Second, possible variability in clinical or data capture practices across different hospitals might affect the validity of EHR data and trends.

Strengths of this study include the use of multiple data sources; the detailed epidemiologic information provided in the population-based EIP surveillance; the inclusion of two widely used EHR systems representing a large number of U.S. acute-care hospitals; and the use of weights to derive national estimates. As has been previously shown with another infection-related condition (sepsis), clinical criteria using EHR data are immune to temporal variations in coding practices that can be significant (*30*), whereas death-certificate data are an insensitive measure of sepsis-related mortality (*31*).

*S. aureus* infections account for substantial morbidity in the United States. Despite significant reductions in health care–associated MRSA infections, progress is slowing. MSSA infections have not decreased as much in hospitals and might be increasing in the community. Adherence to CDC recommendations (*32*) for preventing device- and procedure-associated infections and interrupting transmission, along with innovative interventions tailored to the needs of health care facilities (including decolonization) are needed to further prevent *S. aureus* infections.

**Summary**

**What is already known about this topic?**

Invasive methicillin-resistant *Staphylococcus aureus* (MRSA) infections have been declining in health care settings; however, the rate of decline has recently slowed.

**What is added by this report?**

Nearly 120,000 *Staphylococcus aureus* bloodstream infections and 20,000 associated deaths occurred in the United States in 2017. After years of progress, the rate of decline of MRSA bloodstream infections has slowed, whereas bloodstream infections caused by methicillin-susceptible *S. aureus* are increasing slightly in the community (3.9% annually, 2012–2017).

**What are the implications for public health practice?**

Adherence to CDC recommendations for preventing device- and procedure-associated infections and interrupting transmission, along with innovative, tailored interventions (including decolonization) are needed to further prevent *S. aureus* infections.

## Footnotes

* California (three counties), Connecticut (statewide), Georgia (eight counties), Minnesota (one county), New York (one county), and Tennessee (one county).
